# Scurfy Mice Develop Features of Connective Tissue Disease Overlap Syndrome and Mixed Connective Tissue Disease in the Absence of Regulatory T Cells

**DOI:** 10.3389/fimmu.2019.00881

**Published:** 2019-04-24

**Authors:** Osman K. Yilmaz, Stefanie Haeberle, Meifeng Zhang, Marvin J. Fritzler, Alexander H. Enk, Eva N. Hadaschik

**Affiliations:** ^1^Department of Dermatology, University of Heidelberg, Heidelberg, Germany; ^2^Mitogen Advanced Diagnostics Laboratory, Cumming School of Medicine, University of Calgary, Calgary, AB, Canada; ^3^Department of Dermatology, University Hospital of Essen, Essen, Germany

**Keywords:** mixed connective tissue disease, overlap syndrome, regulatory T cells, scurfy mouse, anti-nuclear antibodies, skin autoimmunity

## Abstract

Due to a missense mutation in the *Foxp3* gene, scurfy mice are deficient in functional regulatory T cells (Treg). The consequent loss of peripheral tolerance manifests itself by fatal autoimmune mediated multi-organ disease. Previous studies have outlined the systemic inflammatory disease and demonstrated production of anti-nuclear antibodies (ANA) in scurfy mice. However, specific autoantibody targets remained to be defined. ANA are immunological markers for several connective tissue diseases (CTD) and target a large number of intracellular molecules. Therefore, we examined scurfy sera for the presence of different ANA specificities and further assessed the organ involvement in these animals. Indirect immunofluorescence was used as a screen for ANA in the sera of scurfy mice and dilutions of 1/100 were considered positive. Addressable laser bead immunoassays (ALBIA) were used to detect specific autoantibody targets. Subsequent histological tissue evaluation was verified by hematoxylin and eosin (H&E) staining. In our study, we observed that nearly all scurfy mice produced ANA. The most prevalent pattern in scurfy sera was nuclear coarse speckled, also known as the AC-5 pattern according to the International Consensus on ANA Patterns. U1-ribonucleoprotein (U1RNP) was found to be the most common target antigen recognized by autoantibodies in scurfy mice. Additionally, scurfy mice exhibited a mild myositis with histological characteristics similar to polymyositis/dermatomyositis. Myopathy-specific autoantibody profile revealed significantly increased levels of anti-SMN (survival of motor neuron) as well as anti-Gemin3 antibodies in scurfy sera. Overall, we demonstrate that the impaired peripheral tolerance in the absence of regulatory T cells in scurfy mice is associated with features of mixed connective tissue disease (MCTD). This includes, along with our previous findings, very high titers of anti-U1RNP antibodies and an inflammatory myopathy.

## Introduction

Scurfy mice are characterized by a complete functional deficiency of regulatory T cells (Treg) due to an X-linked frameshift mutation in the *Foxp3* gene, resulting in an impairment of peripheral tolerance in hemizygous males. The consequent lymphoproliferative disorder is associated with lethal inflammatory multi-organ failure affecting markedly the skin, lungs, kidneys, and liver (1–4). The lack of CD4^+^ FoxP3^+^ Treg leads to an abolished suppression of autoreactive CD4^+^ T cells, which infiltrate numerous organs and cause, along with other inflammatory cells, tissue destruction. B cells also significantly contribute to the autoimmune pathology in scurfy mice via T cell-dependent production of autoantibodies, such as antinuclear antibodies (ANA) (5–7). However, neither the broad spectrum of ANA nor the clinical associations of certain ANA with distinct clinical phenotypes in scurfy mice have been precisely characterized yet.

Connective tissue diseases include a heterogenous group of systemic autoimmune rheumatic disorders including, inter alia, systemic lupus erythematosus (SLE), systemic sclerosis (SSc) and polymyositis/dermatomyositis (PM/DM). Additionally, mixed connective tissue disease (MCTD) as a distinct clinical entity includes some of the clinical features of these three disorders along with high levels of anti-U1RNP (U1-ribonucleoprotein) antibodies (8). The exact pathomechanism underlying these connective tissue disorders (CTD) are still enigmatic. We have previously shown that Treg-deficient scurfy mice exhibit SLE-like autoimmune features such as arthritis, pneumonitis, and nephritis as well as anemia and lymphopenia (7). In line with this observation, we have recently focused on the development of sclerodermatous skin manifestations in scurfy mice by demonstrating that lack of functional Treg in scurfy mice leads also to elevated levels of cutaneous collagen and an inflammatory response as it is partly found in SSc (9). As a crucial step for differential diagnosis of these autoimmune disorders, we determined specific subtypes of ANA in scurfy sera against the following targets: U1RNP, dsDNA (double stranded DNA), histone, Jo-1 (histidyl tRNA synthetase), ribosomal P protein, Sm (U2-U6 RNP), Scl-70 (topoisomerase I), PM-Scl (exosome complex), CENP-B (centromere protein B), PCNA (proliferating cell nuclear antigen), SSA/Ro60, Ro52/TRIM21 (tripartite motif proteins), SSB/La, centromere, RNA polymerase III, Rpp25, and Rpp38 (Th/To complex). We further examined for additional organ involvement which occurs in the absence of regulatory T cells. We identified major features of mixed CTD in scurfy mice including very high titers of anti-U1RNP antibodies and myositis with associated autoantibodies.

## Materials and Methods

### Mice

Female heterozygous *B6.Cg-Foxp3*^*sf*^*/J* (Scurfy) mice were acquired from Jackson Laboratories (Bar Harbor, ME, USA) and bred to male *C57BL/6 wild-type* (WT) mice to generate hemizygous male *B6.Cg-Foxp3*^*sf*^/*Y* (Scurfy) offspring. All mice were held under specific pathogen-free conditions in the animal facilities of the Interfaculty Biomedical Facility (IBF), University of Heidelberg, Germany. Tissues and samples taken from animals were in accordance with the animal protocol (T13/16 and T58/16), approved by the Interfaculty Biomedical Facility of Heidelberg University, Germany.

### Detection of Anti-nuclear Antibodies (ANA)

Serum samples taken from scurfy and WT mice were screened for the presence of anti-nuclear antibodies by indirect immunofluorescence (IIF) assay at dilutions ranging from 1:10 to 1:3200 in PBS with 0.2% Tween 20 (Roth, Karlsruhe, Germany) on human epithelial cells (HEp-20-10) together with primate liver tissue (Euroimmun GmbH, Lübeck, Germany). The slides were incubated for 30 min at room temperature (RT) and washed with PBS-Tween for 5 min. Goat anti-mouse IgG Alexa Fluor 488 (4 μg/ml, Invitrogen, Carlsbad, CA, USA) was added to the slides as secondary antibody. Following an additional incubation phase and subsequent washing, the slides were fitted with cover slips by using Dako Fluorescent Mounting Medium (Dako, Carpinteria, CA, USA). IIF images were obtained with a fluorescence microscope (Zeiss Axioscop 40, Carl Zeiss, Göttingen, Germany) and analyzed as follows: Samples with present fluorescence at a dilution of 1:100 were considered ANA positive. Specific ANA patterns were identified and classified according to the International Consensus on ANA Patterns (ICAP) (10) (https://anapatterns.org/). For semiquantitative analysis, the fluorescence intensity at different dilutions was scored and the results were assigned to a respective antibody titer, as recommended by the manufacturer.

### Screening for ANA Specificities

Multiplexed addressable laser bead immunoassays (ALBIA) provided by TheraDiag (CTD-13 profile, Paris, France) and Inova Diagnostics Inc. (San Diego, CA, USA) were used to identify the specific ANA targets which included: U1RNP, dsDNA, histone, Jo-1, ribosomal P protein, Sm, Scl-70, PM-Scl, CENP-B, PCNA, SSA/Ro60, Ro52/TRIM21, SSB/La, centromere, RNA polymerase III, Th/To-Rpp25, and Th/To-Rpp38. Twenty microliter of suspended beads, 25 μl of sample diluent (Inova Diagnostics Inc.) and 5 μl of diluted mouse serum were added into the wells of 96-well plate. The plate was incubated with agitation at 600 rpm for 30 min at RT, followed by incubation in goat anti-mouse IgG phycoerythrin conjugated secondary antibody (0.5 μg/ml, Jackson ImmunoResearch Lab. Inc.) for 30 min and 600 rpm in the dark. The antibodies to survival of motor neuron (SMN), Gemin3 (DEAD box RNA helicase), Mup44/NT5c1A (cytosolic 5-nucleotidase 1A), and RUVB1/2 (AAA+ ATPases) were detected using a laboratory developed test as previously described (11). Plates were analyzed by using a Luminex-100 plate reader (Luminex Corp., Austin, TX, USA). Cutoff values were established on negative and positive controls in each run and was set at three standard deviations (SD) above the mean for WT mice.

### Histological Analysis of Muscle Tissue

In a routine necropsy, quadriceps muscles were taken from scurfy and WT littermates on day 21 of life for histological evaluation. Muscle tissue was fixed in 4% neural buffered formalin at 4°C overnight and then embedded in paraffin. Five micrometer thick sections were cut and stained with hematoxylin and eosin (H&E). The slides were put in random order and assessed by two independent researchers. Inflammation was scored on the following grading scale: Grade 0: within normal limits, no evidence of inflammation; Grade 1: mild inflammation with few perivascularly scattered lymphocytes in a single focus, Grade 2: moderate inflammation indicating increased numbers of lymphocytes with multifocal distribution; Grade 3: marked inflammation characterized by significantly increased numbers of lymphocytes and several degenerative areas of more striking inflammation or necrotic muscle fibers; Grade 4: severe lymphocytic inflammation indicating diffuse infiltrates with higher extent of necrotizing changes.

### Histological Analysis of Skin Inflammation

For a general assessment of the skin pathology, ears and tails were obtained from Scurfy und WT mice, processed and stained with H&E as described in *1.4*. Skin inflammation was evaluated according to a grading system previously described (12).

### Statistical Analysis

Results are expressed as mean ± SD if not indicated otherwise. Differences were analyzed by two-tailed unpaired *t*-test with Welch's correction. Significance was determined using Prism (GraphPad Software, La Jolla, USA) and *p* < 0.05 were considered significant. (^*^) represents *p* < 0.05, (^**^) represents *p* < 0.01, (^***^) represents *p* < 0.001 and (^****^) represents *p* < 0.0001.

## Results

### IIF Analysis Revealed Predominantly the Nuclear Coarse Speckled Pattern in Scurfy Sera

**Figure 1:**
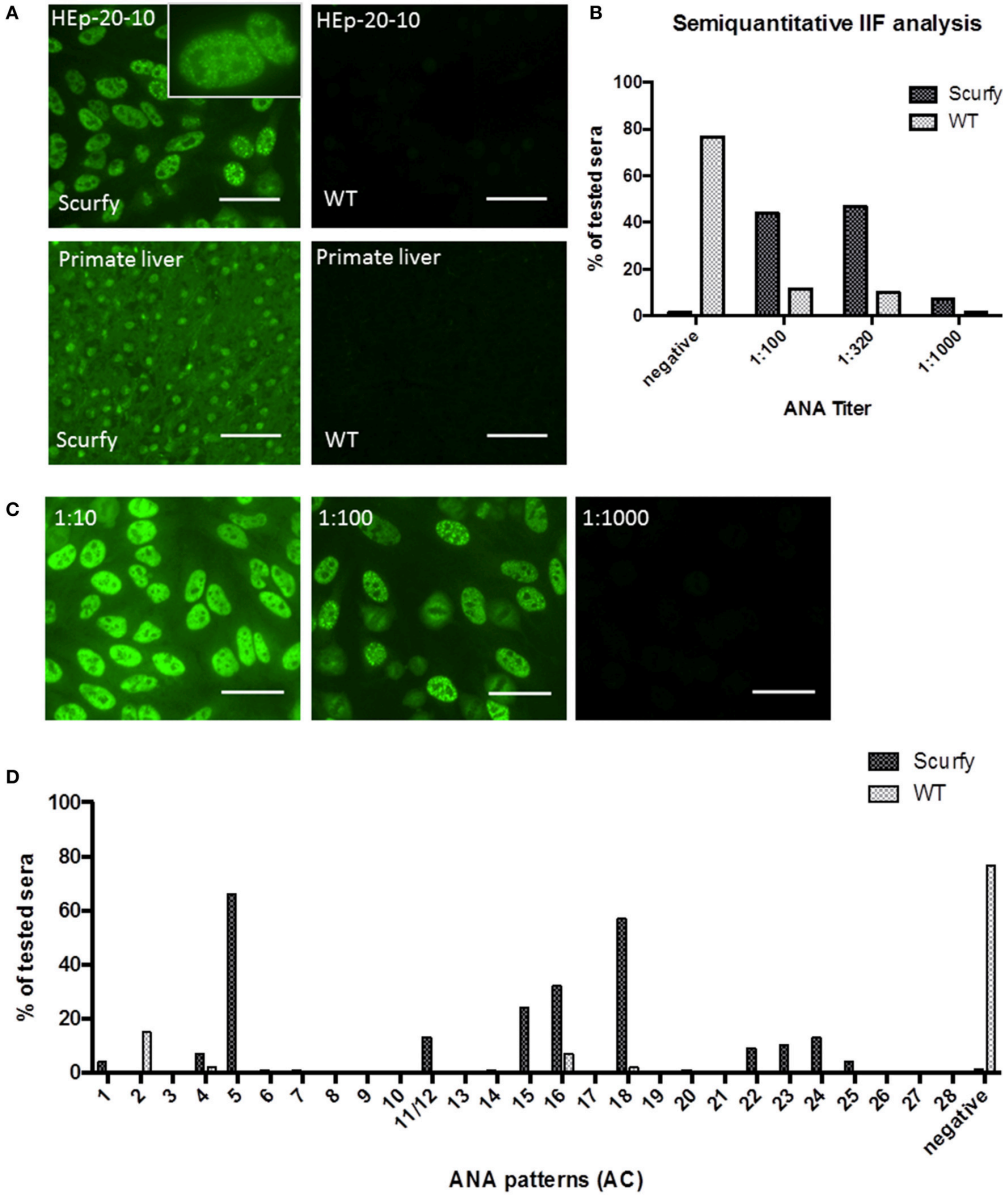
IIF analysis reveals high titers of anti-nuclear antibodies (ANA) in scurfy sera. **(A)** Representative pictures of IIF on HEp-20-10 cells and primate liver tissue with a nuclear coarse speckled pattern in scurfy (left panel) and a negative result in WT (right panel) serum, original magnification 200X, Scale bars represent 50 μm. **(B)** Overview of semiquantitative IIF analysis with ANA titers in scurfy and WT sera. **(C)** IIF images of a scurfy serum in different dilutions for semiquantitative analysis, original magnification 200X, Scale bars represent 50 μm. **(D)** Summary of ANA patterns observed in scurfy and WT sera. Consecutive numbering from anti-cell (AC)-1 to AC-28 is based on the ICAP nomenclature (10) (scurfy *n* = 68, WT *n* = 60).

ANA comprise antibodies directed against intracellular molecules and that are historically linked to several autoimmune disorders including SLE, SSc, PM/DM, and MCTD. As a general diagnostic approach, we initially conducted an ANA screen of scurfy and WT control sera using IIF microscopy on HEp-20-10 cells and primate liver tissue (Figure 1A). Nearly all scurfy sera (98.53%) showed ANA positivity, whereas WT sera were more commonly negative (76.67%) (Figures 1A,D). Further analysis with different dilutions revealed significantly higher ANA titers in scurfy sera compared to WT controls (Figures 1B,C).

Specific targets of ANA are key biomarkers that assist clinicians to distinguish between different autoimmune disease entities, many of which have overlapping clinical presentations. To this end, we identified the IIF patterns in ANA positive sera according to the nomenclature recently established by ICAP (10). Interestingly, the majority of scurfy sera [*n* = 45 out of 68 (66.18%)] showed mainly a nuclear coarse speckled IIF pattern, also referred to as the ICAP AC-5 pattern. Within the small minority of ANA positive WT mice, the nuclear dense fine speckled pattern (AC-2) was the staining pattern predominantly observed (Figure 1D).

### Further Investigations Identified U1RNP as the Most Common Target Antigen by ANA in Scurfy Mice

**Figure 2:**
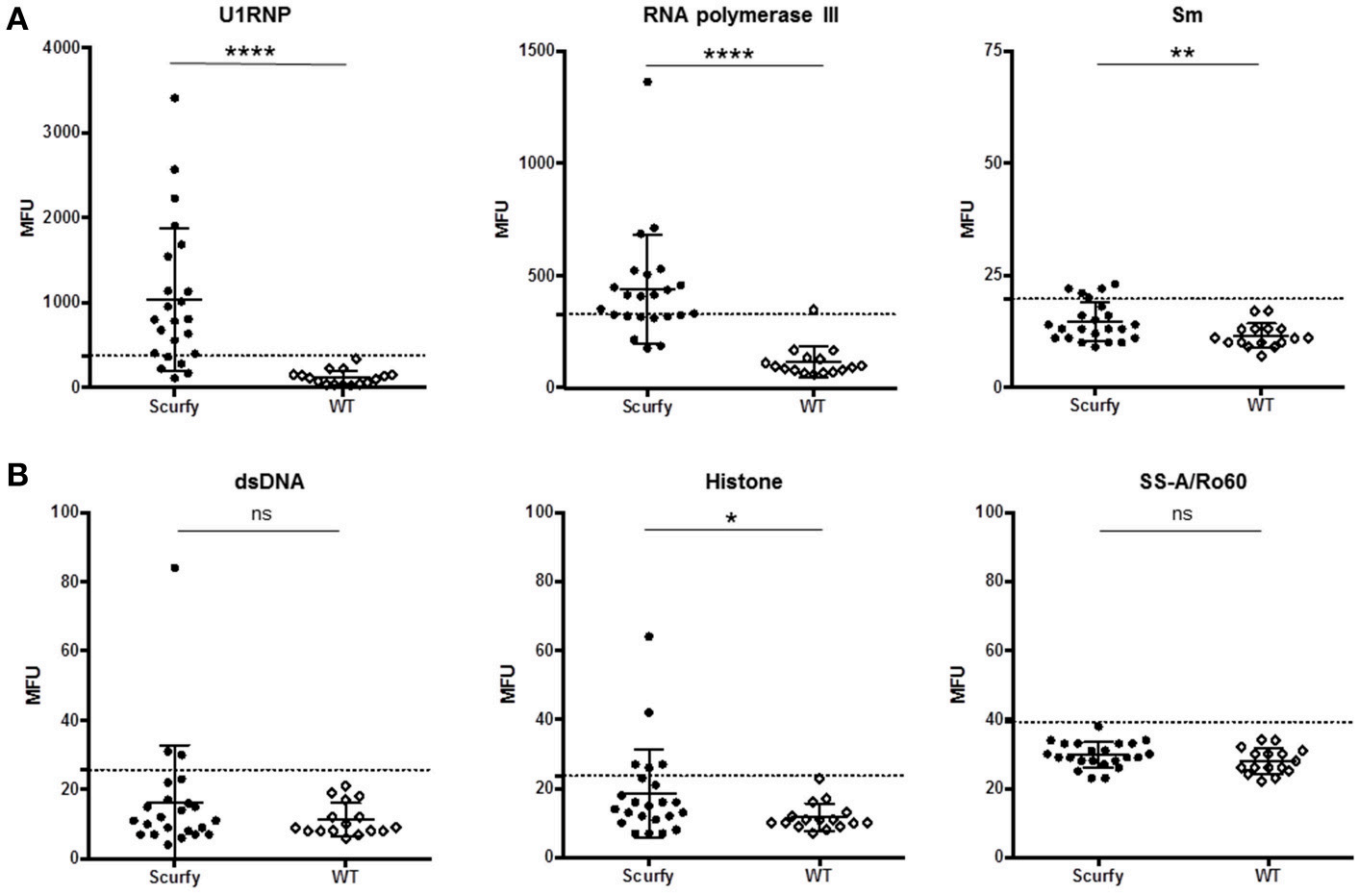
Analysis of specific autoantibodies against CTD associated autoantigens in scurfy and WT sera. **(A)** Detailed examination of AC-5 associated autoantibodies in scurfy and WT sera using ALBIA. **(B)** Summary of lupus-related autoantibodies detected with the same technique. Values are expressed in median fluorescent units (MFU). Dashed lines represent cutoff values established at three SD over the mean of WT controls. (scurfy *n* = 23, WT *n* = 16) (mean ± SD, two-tailed unpaired *t*-test with Welch's correction, ^*^p < 0.05, ^**^p < 0.01, ^****^p < 0.0001).

The pattern code AC-5 is associated with various well-known autoantibodies such as U1RNP and Sm (U2-U6 RNP). In order to elucidate which ANA were specifically associated with this particular pattern in these mice, ALBIA was used as a follow-up assay. In this context, we performed a detailed analysis of a large number of ANA targets, including extractable nuclear antigens (ENA) and other SARD (systemic autoimmune rheumatic disease)-related antigens (Table 1, Supplementary Material). The most striking result to emerge from this analysis was the very high frequency and expression of anti-U1RNP antibodies in scurfy sera. 69.5% (*n* = 16 out of 23) of scurfy mice exhibited positive anti-U1RNP antibodies. A similar, but less marked trend was noted for anti-RNA polymerase III antibodies with positive levels in almost half of the scurfy sera. By comparison, all but one of the analyzed scurfy sera had normal ranges of anti-Sm antibodies (Figure 2A). Surprisingly, no significant difference between scurfy and WT sera was observed in terms of other autoantibodies such as anti-dsDNA and SSA/Ro60 antibodies. Only anti-histone antibodies were found to be produced in higher frequency in scurfy mice, however at a comparatively low significance level (Figure 2B).

**Table 1:** Serological profile of ENA and scleroderma-related autoantibodies in scurfy and WT mice.

**Antibody against/associated with**	**Scurfy mice (*****n*** **=** **23)**		**WT mice (*****n*** **=** **16)**		**Cut-off**	***p*-value**
	**MFU**	**±**	**MFU**	**±**	**MFU**	
U1RNP	1,034 ± 842.2	18/5	119 ± 86.59	0/16	378.77	^****^
Scl-70	37.22 ± 14.74	12/11	20.44 ± 3.52	0/16	31	^****^
RNA polymerase III	438.2 ± 243.1	14/9	115.9 ± 70.7	1/15	328	^****^
Sm	14.65 ± 4.36	5/18	11.50 ± 2.73	0/16	19.7	^**^
CENP-B	11.26 ± 3.97	2/21	8.19 ± 2.88	0/16	16.83	^**^
Ro52/TRIM21	25 ± 7.9	3/20	18.81 ± 7.13	1/15	38.2	^**^
Centromere	45.04 ± 35.17	13/10	23.81 ± 3.92	0/16	35.57	^**^
Th/To–Rpp38	51.04 ± 16.39	0/23	33.06 ± 20.97	1/15	95.97	^**^
Histone	18.48 ± 12.94	5/18	11.69 ± 4	0/16	23.68	^*^
PM/Scl	10.13 ± 5.53	3/20	6.81 ± 3.41	0/16	17.04	^*^
SSB	28.35 ± 8.88	3/20	23.69 ± 3.42	0/16	33.95	^*^
dsDNA	16.3 ± 16.47	3/20	11.25 ± 4.8	0/16	25.64	ns
Jo-1	13.61 ± 7.6	1/22	10.75 ± 7.5	1/15	33.24	ns
Ribosome	41.22 ± 6.05	0/23	40.56 ± 7.78	0/16	63.9	ns
Sm-RNP	19.87 ± 2.18	0/23	18.19 ± 3.29	0/16	28.06	ns
PCNA	9.87 ± 3.09	0/23	8.81 ± 2.48	0/16	16.26	ns
SSA/Ro60	29.74 ± 3.67	0/23	27.94 ± 3.75	0/16	39.19	ns
Th/To—Rpp25	38.83 ± 21.95	1/22	28.5 ± 17.64	0/16	81.42	ns

*Values are expressed in median fluorescence units (MFU). Two-tailed unpaired t-test with Welch's correction*,

* *p < 0.05*,

** *p < 0.01*,

**** *p < 0.0001*.

### Scurfy Mice Showed an Inflammatory Myopathy With Associated Autoantibodies

**Figure 3:**
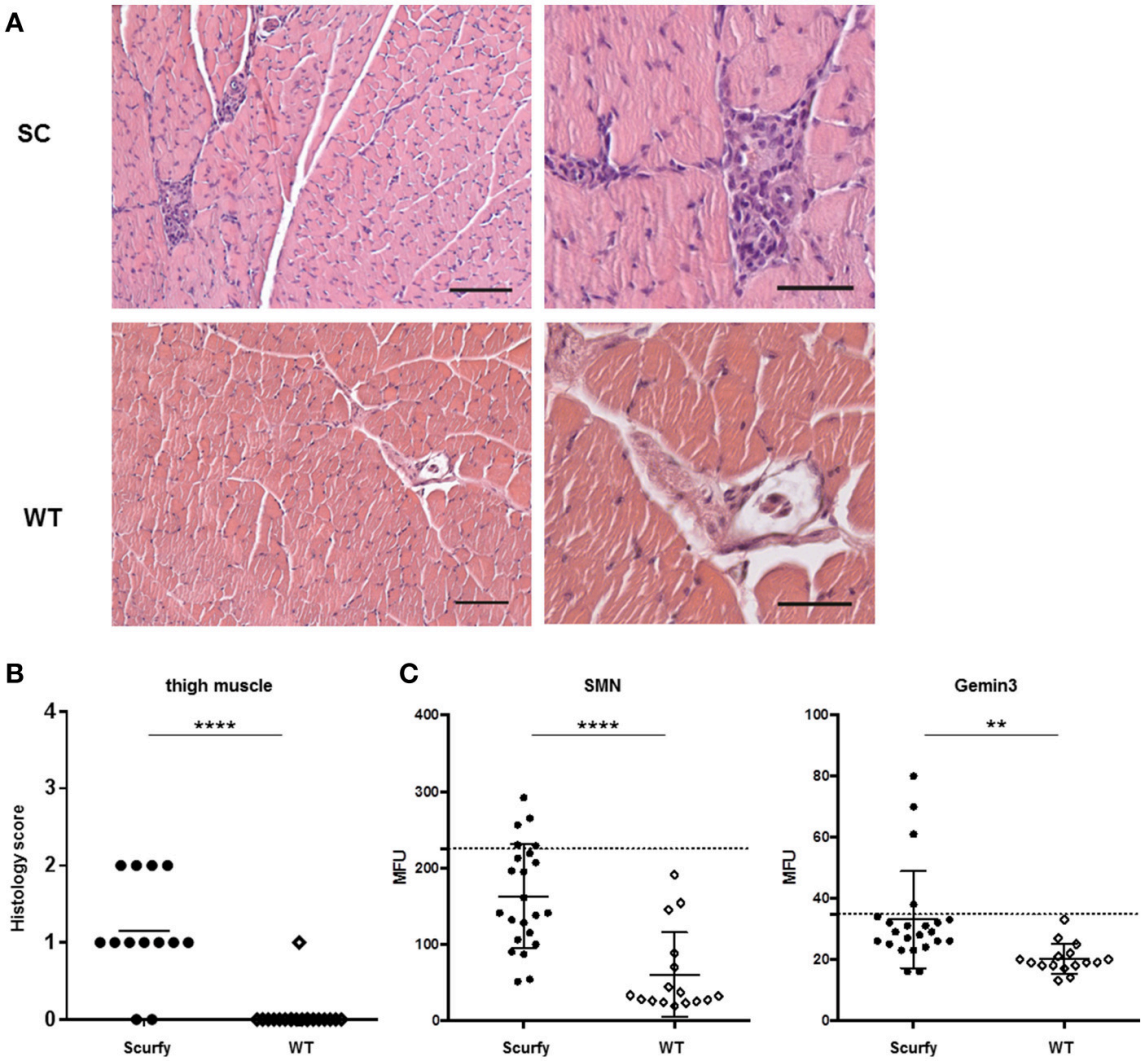
H&E staining exhibits mild myositis in scurfy muscle. **(A)** H&E staining of thigh muscle of scurfy and WT mice, original magnification of 100X (left panels) and 200X (right panels). Scale bars represent 50 and 100 μm, respectively. **(B)** Grading of muscle inflammation in scurfy and WT mice (scurfy *n* = 13, WT *n* = 19). **(C)** Analysis of anti-SMN as well as anti-Gemin3 autoantibodies in scurfy and WT sera using ALBIA. Values are expressed in MFU. Dashed line indicates the cutoff value established at three SD over the mean of WT sera (scurfy *n* = 23, WT *n* = 16) (mean ± SD, two-tailed unpaired *t*-test with Welch's correction, ^**^p < 0.01, ^****^p < 0.0001).

Since anti-U1RNP antibody is a required serological marker for the classification of MCTD (8, 13), we next aimed to verify the clinical resemblance of the scurfy phenotype to MCTD by further detailed analysis of organ involvement in the scurfy mice. The MCTD classification criteria proposed by Sharp et al. is composed of certain clinical features of SLE, SSc, and PM/DM (8). We have previously shown that scurfy mice have SLE and SSc associated features (7, 9) and we next focused on signs of muscle pathology in scurfy mice. Muscle tissue of scurfy mice revealed mild to moderate inflammatory infiltrates characterized by endomysial mononuclear cell infiltrates with invasion of non-necrotic muscle fibers. The inflammatory cells were most prominently distributed in the perivascular area. No signs of perifascicular atrophy or fiber necrosis were detected. Briefly, the overall histological pattern in scurfy muscle showed the main characteristics of limited PM/DM (Figures 3A,B). Furthermore, the myopathy-specific autoantibody profile highlighted elevated levels of anti-SMN as well as anti-Gemin3 antibodies in scurfy sera (Figure 3C), whereas antibodies against Jo-1, PM/Scl, Mup44/NT5c1A (14), RUVBL1/2 (15) as detected by ALBIA were not significantly increased (Table 2).

**Table 2:** Serological profile of myopathy-related autoantibodies in scurfy and WT mice.

**Antibody against/associated with**	**Scurfy mice (*****n*** **=** **23)**		**WT mice (*****n*** **=** **16)**		**Cut-off**	***p* value**
	**MFU**	**±**	**MFU**	**±**	**MFU**	
SMN	162.9 ± 68.44	5/18	60.38 ± 54.92	0/16	225.14	^****^
Gemin3	33.04 ± 15.90	4/19	20.19 ± 4.88	0/16	34.82	^**^
Mup44/NT5c1A	205.1 ± 46.19	3/20	186.4 ± 15.15	0/16	231.85	ns
RUVBL1	594.9 ± 109.4	3/20	555.9 ± 69.75	0/16	765.15	ns
RUVBL2	471.6 ± 82.93	0/23	463.6 ± 54.58	0/16	627.34	ns

*Values are expressed in median fluorescence units (MFU). Two-tailed unpaired t-test with Welch's correction*,

** *p < 0.01*,

**** *p < 0.0001*.

### Dermatopathological Analysis of Scurfy Ear and Tail Showed a Severe Lymphohistiocytic Inflammation

**Figure 4:**
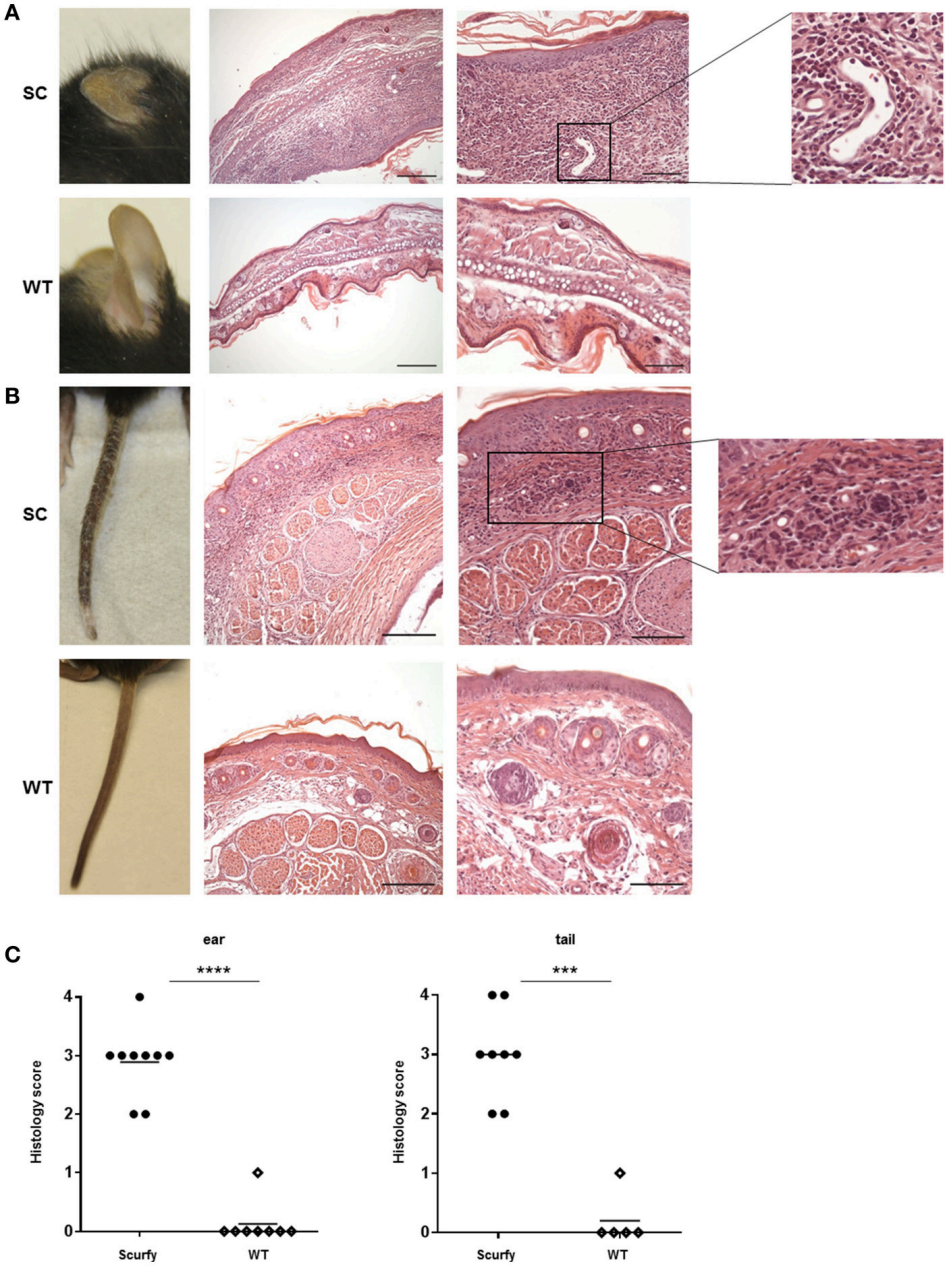
Histological analysis of scurfy skin shows strong resemblance to typical findings in MCTD. **(A)** Macroscopic (left panel) and histological (right panels) evaluation of ears obtained from scurfy and WT mice. **(B)** Macroscopic (left panel) and microscopic (right panels) analysis of tails of scurfy and WT mice. **(C)** Grading of the inflammation status in ears and tails of scurfy mice in comparison to WT controls. (scurfy *n* = 9, WT *n* = 8 for the ear and scurfy *n* = 8, WT *n* = 5 for the tail) (mean ± SD, two-tailed unpaired *t*-test with Welch's correction, ^***^p < 0.001, ^****^p < 0.0001).

In order to determine if scurfy mice develop a MCTD-like cutaneous phenotype, we closely monitored the inflammation pattern in the ear and tail of scurfy mice. Macroscopic examination on day 21 demonstrated severe changes in both organs. Compared to T mice, ear skin of scurfy mice appeared substantially smaller and thicker with significant scaliness (Figure 4A). Scurfy tail showed a patchy inflammation on the skin, comprising encrusted areas and erythematous erosions in succession. Advanced stages of inflammation indicated also gangrenous changes (Figure 4B). Histological analysis of H&E-stained ear and tail sections confirmed the marked thickness and showed massive infiltration of pleomorphic mononuclear cells, especially in the dermoepidermal junction (interface dermatitis) and dermis. Perivascular or subcutaneous extension of the inflammation as well as vascular ectasia were occasionally observed. The infiltrates were mainly of lymphohistiocytic character. In both ear and tail of scurfy mice, a remarkable hyperkeratosis was detected (Figures 4A–C).

## Discussion

Treg represent a lineage of T cells which play a fundamental role in maintaining humoral tolerance in the periphery. This subset of “suppressor T cells” is identified as FoxP3-expressing CD4^+^ T cells (16, 17). The unrestrained expression of FoxP3 is essential for the development and function of Treg (4). Accordingly, a disruption of the *Foxp3* gene in scurfy mice results in an autoimmune lymphoproliferative disorder with fatal multi-organ inflammation (18). Since the causative mutation occurs in orthologous genes, the scurfy phenotype is indicated as the murine equivalent of the human IPEX syndrome (immune dysregulation, polyendocrinopathy, enteropathy, X-linked) (3, 19). The consequent autoimmune pathology is not only coordinated by T cells, but also B cells provide contribution through antibody production, such as ANA (6, 20).

ANA are diagnostic hallmarks of SARD and directed against several intracellular components which tend to be ubiquitously expressed in many cells and tissues (21). To detect autoantibodies against SARD-associated antigens, we performed an IIF analysis. In agreement with previous results, we found significant ANA positivity and elevated ANA titers in scurfy sera compared to WT controls. We subsequently highlighted that scurfy sera predominantly revealed the AC-5 pattern in the IIF analysis, whereas the most frequent pattern of WT sera was the AC-2 pattern. It is interesting to note that the AC-2 pattern is associated with anti-DFS70/LEDGF (dense fine speckled/lens epithelium-derived growth factor) antibodies which have been described as exclusion markers for SARD (22, 23). Additional tests showed that the most striking target antigen in scurfy sera was U1RNP, the major serological marker for MCTD. But there is also evidence that some scleroderma-associated autoantibodies such as anti-RNA polymerase III and anti-Scl-70 antibodies are expressed at higher levels in scurfy sera. Only a minority of the tested scurfy sera showed lupus-related autoantibodies, including anti-dsDNA and anti-SSA/Ro60. Taken together, the serological profile of scurfy mice indicates striking correspondence to a CTD overlap syndrome, including the serological hallmark of MCTD.

The concept of MCTD as a distinct clinical entity has existed for more than 40 years. However, there is still some controversy surrounding classification criteria or relationship to other CTD (24, 25). One of the proposed criteria sets was delineated by Sharp with high titers of anti-U1RNP antibodies, Raynaud's phenomenon, swollen hands and elements of SLE, scleroderma and PM/DM (8). It is worth noting that the whole CTD spectrum is subject to fluctuations and clinical changes. In this regard, there are reports addressing that, in many cases, MCTD evolves into other diseases from this spectrum (26, 27). This “phenotypic shift” can for instance lead to development of glomerulonephritis and production of anti-dsDNA antibodies [reviewed in (28)]. This is in accord with our observation that only a few scurfy mice produced anti-dsDNA antibodies. Further research should be undertaken to analyze longitudinal clinical and serological changes that occur during the course of disease in scurfy mice.

The exact role of anti-U1RNP in the pathogenesis of MCTD is still poorly understood. Recent evidence indicates a strong and distinct HLA (human leukocyte antigen)-association with MCTD which seems to suggest that HLA-restricted T cell subsets might be responsible for induction of antibody production by B cells [(29–32); reviewed in (28, 33)]. Accordingly, an HLA-DR4 transgenic mouse model developed MCTD-like lung disease upon immunization with U1RNP and Freund's complete adjuvant (34). Another study reported further insight into the immunology of MCTD by noting decreased levels of naturally occurring Treg in MCTD patients (35).

We have previously demonstrated that scurfy mice develop lupus-like clinical features such as pneumonitis, synovitis and mesangioproliferative glomerulonephritis. Moreover, hematological abnormalities included anemia and lymphopenia, although thrombocytopenia was not observed (7). In a recent publication we reported that the scurfy phenotype closely resembles scleroderma as scurfy skin has a higher collagen content and the inflammatory response is driven by CD4^+^ T cells with Th2 differentiation and alternatively-activated (M2) macrophages (9). Consistent with these findings, in the present study we show that scurfy mice also spontaneously develop a limited myositis with a histological pattern similar to PM/DM [reviewed in (36)]. This concurs fairly well with previous observations of mild inflammatory myopathy in MCTD (8, 37). As reported by Young et al. CD4^+^ T cells from scurfy lymph nodes are also able to induce myositis after transfer into RAG-1-null recipients (38). The same report pointed out the crucial role of Treg in myositis by showing that cotransfer of Treg leads to complete suppression of myositis.

As an additional finding, we identified a significant expression of anti-SMN and anti-Gemin3 antibodies in scurfy sera. Mutation of SMN is well-known to cause a genetic neuromuscular disorder called spinal muscular atrophy (SMA) (39, 40). Gemin3 encodes a DEAD box RNA helicase and is an essential component of the SMN complex (41). Interestingly, Satoh et al reported that antibodies to the SMN complex are an antigenic target recognized by antibodies in patients with PM/SLE overlap and suggested that the SMN complex is fundamental for the assembly of snRNPs (small nuclear ribonucleoproteins) (42). The conceptual linkage of anti-SMN/Gemin autoantibodies with anti-U1RNP was supported in a recent case report of a patient with myositis who developed antibodies to both targets (11). These findings support the hypothesis that autoimmune-mediated inflammatory myopathies and genetic neuromuscular disorders could have common pathways (43). Importantly, MCTD is also associated with neuropsychiatric manifestations which have been found to correlate with intrathecal production of anti-U1RNP antibodies (44). Therefore, further experimental studies are needed to estimate the scurfy phenotype from a neuropathological perspective.

Additional histological experiments demonstrated severe inflammatory cutaneous alterations of scurfy ear and tail. Our findings are in line with previous studies by Hadaschik et al. which focused on the dermatopathology of the scurfy back skin and identified interface dermatitis and significant lymphohistiocytic infiltrates, indicating lupus-like histological changes. Their results also revealed IgG deposits in the dermoepidermal junction of the scurfy back skin similar to lupus band in patients with SLE (7). Although there are already multiple criteria sets for the diagnosis of MCTD, only few researchers have addressed the question of dermatopathological changes in MCTD patients. A remarkable study demonstrated that the skin pathology in MCTD bore a strong resemblance to that of subacute cutaneous lupus erythematosus (SCLE), including interface dermatitis (75% of the patients) and positive lupus band test (37.5% of the patients). MCTD led also to a vasculopathy paralleling that observed in skin lesions of dermatomyositis (45).

Taken together, our findings suggest that the scurfy phenotype reveals clinical and serological features of a CTD overlap syndrome akin to MCTD, including high titers of anti-U1RNP antibodies and myositis. Nevertheless, we do not characterize the scurfy mouse as an MCTD model, since it develops many other autoimmune features like nephritis or hepatitis. Our results mainly strengthen the hypothesis that Treg deficiency and the subsequent loss of immune homeostasis lead to development of serological and pathological features of overlap CTD, including MCTD.

## Ethics Statement

Tissues and samples taken from animals were in accordance with the animal protocol (T13/16 and T58/16), approved by the Interfaculty Biomedical Facility of Heidelberg University, Germany.

## Author Contributions

OY, SH, MF, EH, and AE conceived the project and experiments. OY, SH, and MZ performed the experiments and statistical analysis. OY wrote the first draft of the manuscript. SH and MZ wrote sections of the manuscript. All authors contributed to manuscript revision, reviewed, and approved the submitted version.

### Conflict of Interest Statement

MF is a consultant to and has received honoraria from Inova Diagnostics (San Diego, CA, USA) and Werfen International (Barcelona, Spain). The remaining authors declare that the research was conducted in the absence of any commercial or financial relationships that could be construed as a potential conflict of interest.

## Abbreviations

ANA: anti-nuclear antibody
CTD: connective tissue diseases
ENA: extractable nuclear antigens
MCTD: mixed connective tissue disease
PM/DM: Polymyositis/dermatomyositis
SARD: systemic autoimmune rheumatic disease
SCLE: subacute cutaneous lupus erythematosus
SLE: systemic lupus erythematosus
SMN: survival of motor neuron
SSc: systemic sclerosis
Treg: regulatory T cells
U1RNP: U1-ribonucleoprotein.
