# Supplementary material for: Scurfy Mice Develop Features of Connective Tissue Disease Overlap Syndrome and Mixed Connective Tissue Disease in the Absence of Regulatory T Cells
Source: Front Immunol. 2019 Apr 24;10:881. doi: 10.3389/fimmu.2019.00881 (PMC6491778; doi:10.3389/fimmu.2019.00881)
Supplement: Supplementary file 1 [file Table_1.DOCX]

Supplementary Material

# Supplementary Table

# Supplementary Table 1: Antibody profile of a single scurfy mouse compared to a WT mouse. Values are expressed in median fluorescent units (MFU). Cutoff values are established at three SD over the mean of WT controls.

| **Antibody against/ associated with** | **Scurfy mouse** | | **WT mouse** | | **Cut-off** |
| --- | --- | --- | --- | --- | --- |
|  | **MFU** | **+/-** | **MFU** | **+/-** | **MFU** |
| U1RNP | 2565 | + | 113 | - | 378.77 |
| Scl-70 | 40 | + | 23 | - | 31 |
| RNA polymerase III | 687 | + | 111 | - | 328 |
| Sm | 21 | - | 13 | - | 19.7 |
| CENP-B | 10 | - | 12 | - | 16.83 |
| Ro52/TRIM21 | 28 | - | 20 | - | 38.2 |
| Centromere | 33 | - | 34 | - | 35.57 |
| Th/To – Rpp38 | 48 | - | 23 | - | 95.97 |
| Histone | 14 | - | 11 | - | 23.68 |
| PM/Scl | 8 | - | 11 | - | 17.04 |
| SSB | 24 | - | 32 | - | 33.95 |
| dsDNA | 7 | - | 18 | - | 25.64 |
| Jo-1 | 27 | - | 4 | - | 33.24 |
| Ribosome | 46 | - | 50 | - | 63.9 |
| Sm-RNP | 21 | - | 18 | - | 28.06 |
| PCNA | 9 | - | 11 | - | 16.26 |
| SSA/Ro60 | 28 | - | 30 | - | 39.19 |
| Th/To – Rpp25 | 52 | - | 25 | - | 81.42 |
| SMN | 213 | - | 145 | - | 225.14 |
| Gemin3 | 29 | - | 19 | - | 34.82 |
| Mup44/NT5c1A | 296 | - | 195 | - | 231.85 |
| RUVBL1 | 636 | - | 573 | - | 765.15 |
| RUVBL2 | 510 | - | 461 | - | 627.34 |
